# Preventing hepatocellular carcinoma in Egypt: results of a Pilot Health Education Intervention Study

**DOI:** 10.1186/s13104-015-1351-1

**Published:** 2015-08-29

**Authors:** Doa’a A. Saleh, Sania Amr, Irene A. Jillson, Judy Huei-yu Wang, Nancy Crowell, Christopher A. Loffredo

**Affiliations:** Cairo University, Cairo, Egypt; University of Maryland, Baltimore, MD USA; Department of Oncology, Georgetown University, 3970 Reservoir Rd, Washington DC, 20057 USA

**Keywords:** Liver cancer, Prevention, Hepatitis C virus, Aflatoxin, Pesticides

## Abstract

**Background:**

Hepatocellular carcinoma (HCC), one of the most fatal malignancies, is particularly prevalent in Egypt, where we previously found deficiencies in knowledge concerning HCC and its risk factors. Hepatitis B and C viral infections are highly prevalent in Egypt, pesticides are very commonly used, and diets are often contaminated by aflatoxin, especially in rural areas.

**Methods:**

We conducted a study to pilot test a health education intervention addressing HCC, its risk factors, and its main modes of prevention. It included four health education modules: HCC, hepatitis viruses, pesticides and aflatoxin. We used a pre- and post-intervention set of questionnaires to assess knowledge gained by the participants.

**Results:**

A total of 25 participants from a village in the Nile Delta area attended the health education session and completed the questionnaires. The education intervention significantly increased the participants’ knowledge on HCC and its risk factors, particularly regarding the use of pesticides at home and aflatoxin contaminated foods (both p < 0.05). Overall, there was a 12 % increase in the number of participants who believed that HCC could be prevented, and they reported their intention to practice prevention for HCC risk factors.

**Conclusions:**

We found that the education intervention we pilot tested was feasible and proved effective in increasing participants’ knowledge. Future efforts should focus on implementing targeted education programs in high-risk populations in Egypt.

## Background

Hepatocellular carcinoma (HCC), the most common type of primary liver tumor, arises directly from hepatocytes and accounts for about 90 % of all primary hepatic malignancies. HCC is the 5th most common cancer worldwide, with 600,000 new cases each year [1], and the highest rates of HCC are in areas of the world with high rates of chronic hepatitis B virus (HBV) and/or hepatitis C virus (HCV) infections.

Epidemiological studies have established that chronic infection with HBV [2] or HCV [3] precedes the development of HCC. HCV, like HBV, is spread through contact with infected body fluids, but unlike HBV, there is no vaccine to prevent HCV infection. Therefore, HCV as a major cause of HCC will continue to increase unless transmission can be interrupted. In addition, effective treatments that have been available thus far for HCV have serious side effects and a cure rate limited to 25–70 %, depending on the HCV genotype. More recent curative therapies have minor adverse effects but are very expensive, precluding most patients in developing countries from access. In the absence of a vaccine, HCV prevention has focused on infection control, but these efforts are inadequate where advanced health care infrastructure is lacking. In many countries, testing for HBV and HCV is not a routine component of primary care, although HBV vaccination is compulsory in some countries, and this is reducing HCC incidence, especially in high-endemic areas [4].

In Egypt, HCV infection is highly prevalent and is largely attributed to the parenteral antischistosomal therapy campaigns conducted from the 1950s through the 1980s. However, contaminated needles are not the only way of transmitting HCV. Indeed, studies of intra-familial transmission have demonstrated increased rates of parent-to-offspring and sibling-to-sibling HCV transmission in rural communities [5], and one of the predictors of incident HCV infection has been found to be having a family member who tests positive for HCV antibodies [6]. Moreover, a significant challenge to HCV control is the lack of a prevention model that addresses the problem at the community level.

In addition, environmental exposures are known risk factors for HCC. Dietary aflatoxin, i.e. the major toxin AFB1 produced by Aspergillus molds that infect stored grains and other foods, is classified by the International Agency of Research on Cancer as a class 1 human carcinogen [7]. Multiple studies in Egypt have shown the presence of AFB1-albumin adducts in human blood [8, 9], and AFB1 has been detected at moderate to high levels in food samples purchased in Egypt [10]. Agricultural exposures to insecticides and herbicides are common in Egypt, and they were previously reported to increase the risk of HCC [11], although not to the same level of magnitude as with HBV and HCV.

Aside from HBV, which is being prevented and controlled through national vaccination campaigns worldwide [4], there are barriers to the prevention and control of the remaining risk factors. Exposure to pesticides and aflatoxin can be prevented through the implementation of appropriate protective measures, which would be expected to be amenable to education-based interventions. For example, community and individual prevention approaches—including safer application of pesticides and the use of non-chemical alternatives—offer a similar avenue for prevention through education and outreach [12]. Aspergillus mold contamination and the accumulation of AFB1 in foods can likewise be prevented through safe food storage and preparation practices.

We previously conducted a qualitative study to understand the current knowledge and health practices surrounding HCC and its prevention in rural Egypt. We investigated knowledge, attitudes, beliefs, and practices concerning HCV and pesticides in two Egyptian villages. The interviews revealed misconceptions about the modes of transmission of HCV (e.g. polluted air and water) and lack of knowledge about pesticides [13]. Therefore, we developed and pilot tested an education-based intervention that aimed to increase knowledge about HCC and its risk factors in a rural population in northern Egypt, which suffers from high prevalence of HCV and widespread exposure to pesticides and aflatoxins. Our aim in conducting the present study was to assess the feasibility and potential efficacy of an education-based intervention to increase knowledge of HCC, its risk factors, and preventive practices.

## Methods

The Institutional Review Boards of both Georgetown University and Cairo University approved the study procedures.

### Study population

In collaboration with Cairo University, and with the help of the Rural Health Unit Director in the area, we approached participants from one village (approximately 5000 persons) located in the Nile Delta region, which had high prevalence rates of both HCV and HBV infection. The study coordinator visited the rural health unit and randomly examined the available family health records for potential study participants. The inclusion criteria were being a resident of the village, 18–70 years old, and physically and mentally able to participate in an educational intervention program. Social community workers were then asked to visit homes and invite the selected participants to attend the health education session on the assigned day. Thirty percent of the contacted participants did not come to the health unit on the assigned day to participate in the health education session. Thus, a total of 25 subjects agreed to participate and provided informed consent.

### Education-based intervention

We developed four knowledge modules (HCC, viral hepatitis, pesticides, and aflatoxin). We used educational materials from sources including the Centers for Disease Control and Prevention’s Fact Sheets for HBV and HCV [14]; the Environmental Protection Agency’s Citizen’s Guide to Pest Control and Pesticide Safety [15]; and the Workgroup Report on Public Health Strategies for Reducing Aflatoxin Exposure in Developing Countries [16]. We presented the materials for the modules mainly via illustration, rather than text, and an external expert reviewed them before they were translated into Arabic.

We also developed pre-post-intervention tests to assess knowledge gain and asked the participants only after the educational intervention about their intent to change practice. For the latter, we asked yes/no questions about their intent to “properly store food to decrease growth of fungus”, to “use non-chemical ways to keep pests out of the house and away from crops”, to “practice safer ways of handling pesticides”, to “seek medical testing for HBV and HCV”, and to “become involved in community efforts to prevent cancer”. The sample size did not allow for psychometric testing of the instrument. However, we found that reliability testing resulted in a Cronbach’s alpha of 0.74, which indicated adequate total scale reliability.

The educational intervention was conducted by the primary author (a public health physician) in a 4-h presentation attended by the participants. Interviewers administered the pre-intervention test prior to the educational presentation of the four modules described above, and they re-tested the subjects 2 h later using the same instrument (post-intervention assessment).

### Scoring of the tests

A score was created for each of the four modules to show the change in the percentage of correct answers from the pre- to post-intervention. This was done by scoring one point for the correct answer and zero for incorrect or “don’t know” answers. For example for the questions “What causes liver cancer?” the correct answers were hepatitis B and C, pesticides and foods contaminated by fungus; while water pollution and air pollution were incorrect answers. The sum of all the points constituted the final score for each of the modules. A maximum of 7, 5, 14 and 13 points could be obtained in the HCC, HCV, pesticides and aflatoxin modules, respectively. This led to a total of 39 possible points for all of the items in the survey instrument. All scores were expressed as a percentage of the total score (i.e. [score achieved/total possible score] × 100).

### Statistical analysis

We used (1) Wilcoxon signed-ranks test to compare the mean percent scores for each and all the health education modules, pre- and post- intervention; and (2) McNemar’s test to compare the paired pre- and post-intervention yes and no responses.

## Results

### Demographics

Of the 25 participants, 11 were male, and the age range among all participants was 23–64 years (mean 33.5). The age range among male participants in particular was 28–64 (mean 39.4, median 34), and the age range among women was 23–56 (mean 33.6, median 33); no significant difference in age was found between male and female participants (p = 0.196). Twelve participants (48 %) had completed technical or post-technical (but not university) education, while among the remaining subjects six could read but hardly write, four could neither read nor write, and three could barely read and write. Nineteen participants (76 %) worked outside of the home: ten described themselves as laborers, five as professionals (including nurse, social worker, teacher and imam), three as clerks, and one as a farmer.

### Pre- and post-intervention scores of total and individual health education modules

Comparison of the pre- and post-intervention scores for the sample showed a significant increase in the post-intervention mean score for the pesticides and aflatoxin modules separately, as well as for the total score by 17, 33 and 18 % respectively (p < 0.05) (Table 1). Although not statistically significant, the overall scores in the HCC and hepatitis modules increased after the intervention by 20 and 21 %, respectively. The intervention tended to enhance participants’ beliefs that they could protect themselves from developing liver cancer by 20 %, but this did not reach statistical significance (p = 0.06).

**Table 1:** Comparison of pre- and post-intervention mean percent scores for each and all the health education modules

Module	Mean percent score (SD)	Mean percent change (SD)	p-value*
HCC			
Pre-	72.7 (18.6)	19.7 (37.9)	0.09
Post-	81.3 (11.1)		
Hepatitis			
Pre-	76.0 (18.3)	21.0 (48.8)	0.07
Post-	84.0 (12.9)		
Pesticides			
Pre-	72.9 (13.0)	18.0 (47.8)	0.02
Post-	80.6 (9.1)		
Aflatoxin			
Pre-	50.8 (11.3)	33.1 (48.3)	0.001
Post-	63.1 (8.6)		
Total			
Pre-	64.8 (10.0)	17.9 (26.6)	<0.001
Post-	74.0 (4.5)		

* p-value for comparing the mean scores using Wilcoxon Signed-Ranks Test

### Knowledge about HCC and its risk factors

Prior to the intervention, five participants (20 %) had never heard of hepatocellular carcinoma. After the intervention, all participants reported being aware of this health problem. Knowledge that viral hepatitis, pesticides and foods contaminated with aflatoxin are risk factors for HCC showed improvements by 16, 12 and 8 % respectively, albeit none of these increases were statistically significant. Moreover, following the education intervention there was a non-significant increase (84 % in the pre-intervention vs. 96 % in the post-intervention) in the number of participants who believed that HCC can be prevented.

### Knowledge about hepatitis B and C viruses

Prior to the intervention, 16 % (N = 4) of participants had never heard of hepatitis B and C viruses. Immediately after the intervention, all participants reported knowing about these viruses. Correct knowledge about the mode of transmission of the hepatitis viruses increased from 84 % in the pre- to 96 % in the post-intervention; however, those participants who incorrectly believed that polluted water and sewage are also transmission vehicles for HCV and HBV continued to believe so.

### Knowledge about pesticides exposures

After the intervention, participants’ knowledge that pesticides are used to kill insects, weeds and molds increased but not significantly; however, their awareness of harmful exposure to pesticides in the home increased by 28 % (p = 0.016) (Table 1). All participants responded correctly regarding how to protect themselves from pesticides, such as following directions for the proper handling of each pesticide and avoiding being in locations that were recently treated by pesticides. A non-significant increase (12 %) in the belief that using non-chemical means of controlling pests is effective was observed after the intervention (Table 2).

**Table 2:** Comparison of the pre- and post-intervention responses for the pesticides module

Question	Pre-intervention			Post-intervention			P value*
	Yes, N (%)	No, N (%)	Don’t know, N (%)	Yes, N (%)	No, N (%)	Don’t know, N (%)	
Have you ever heard of pesticides?	24 (96)	1 (4)	0	25 (100)	0	0	1.00
What are pesticides?							
Chemicals to kill insects	22 (88)	1 (4)	2 (8)	25 (100)	0	0	0.25
Chemicals to kill weeds	21 (84)	3 (12)	1 (4)	25 (100)	0	0	0.12
Chemicals to wash clothes	13 (52)	11 (44)	1 (4)	18 (72)	7 (28)	0	0.34
Chemicals to kill fungus or mold	15 (60)	6 (24)	4 (16)	21 (84)	3 (12)	1 (4)	0.07
Chemicals to clean floors	16 (64)	8 (32)	1 (4)	18 (72)	7 (28)	0	1.00
How do people become exposed to pesticides?							
Living near a field that has been sprayed	24 (96)	1 (4)	0	25 (100)	0	0	1.00
Applying these chemicals in the house	*18* (*72*)	*7* (*28*)	*0*	*25* (*100*)	*0*	*0*	*0.01*
Applying these chemicals in farming	22 (88)	2 (8)	1 (4)	25 (100)	0	0	0.25
Eating food that was sprayed in the field	24 (96)	1 (4)	0	24 (96)	1 (4)	0	1
How can people protect themselves from pesticides?							
Handle them according to the directions	24 (96)	1 (4)	0	25 (100)	0	0	1.00
Get vaccinated against them	23 (92)	0	2 (8)	20 (80)	4 (16)	1 (4)	0.12
Avoid going to places that were recently treated	24 (96)	0	1 (4)	25 (100)	0	0	1.00
Use non-chemical ways to keep pests away	17 (68)	5 (20)	3 (12)	20 (80)	3 (12)	2 (8)	0.45

* p-value for comparison between pre- and post-intervention responses as “yes, no, or don’t know” using McNemar’s test

Italics indicate statistically significant results (*p* < 0.05)

### Knowledge about aflatoxin exposure

There was substantial knowledge gain with respect to the types of food susceptible to fungal contamination. The percentage of correct answers increased by 52 % regarding knowledge of susceptibility to fungal infestation of rice (p = 0.001) and 32 % for corn (p = 0.008), but only by 20 % for uncooked beans (p = 0.18). The gain in knowledge about preventive measures to inhibit fungus from growing in foods, such as storing foods off the ground and keeping foods dry, did not significantly increase (Table 3).

**Table 3:** Comparison of the pre- and post-intervention responses for the Aflatoxin module

Question	Pre-intervention			Post-intervention			P value*
	Yes, N (%)	No, N (%)	Don’t know, N (%)	Yes, N (%)	No, N (%)	Don’t know, N (%)	
Do you know about the fungus that might affect food?	24 (96)	1 (4)	0	25 (100)	0	0	1.00
What kinds of foods or drinks are susceptible to fungal contamination?							
Cheese	20 (80)	5 (20)	0	20 (80)	5 (20)	0	1.00
Rice	*8* (*32*)	*17* (*68*)	*0*	*21* (*84*)	*3* (*12*)	*1* (*4*)	*0.001*
Peanuts	21 (84)	3 (12)	1 (4)	24 (96)	1 (4)	0	0.37
Oranges	21 (84)	2 (8)	2 (8)	19 (76)	6 (24)	0	0.21
Corn	*17* (*68*)	*7* (*28*)	*1* (*4*)	*25* (*100*)	*0*	*0*	*0.008*
Meat	19 (76)	6 (24)		15 (60)	9 (36)	1 (4)	0.37
Uncooked beans	16 (64)	8 (32)	1 (4)	21 (84)	3 (12)	1 (4)	0.18
Soda	4 (16)	17 (68)	4 (16)	8 (32)	15 (60)	2 (8)	0.79
What can be done to prevent fungus from growing in food?							
Wash fresh fruits and vegetables when we get them home	25 (100)	0	0	25 (100)	0	0	1.00
Store foods off the ground	20 (80)	5 (20)	0	22 (88)	3 (12)	0	0.68
Keep the foods dry	21 (84)	4 (16)	0	24 (96)	1 (4)	0	0.25
Cook foods at a high temperature	18 (72)	7 (28)	0	17 (68)	8 (32)	0	1.00

* p-value for comparison between pre- and post-intervention responses as “yes, no, or don’t know” using McNemar’s test

Italics indicate statistically significant results (*p* < 0.05)

### Intent to use best practices

After attending the educational sessions, and in response to the question regarding their intent to use best practices, all participants indicated their intent to properly store food to decrease growth of fungus, to seek medical testing for HBV and HCV, and to become involved in community efforts to prevent cancer. There was somewhat less commitment to using non-chemical means of controlling pests, with 80 % saying yes to the use of non-chemical means and 92 % saying yes to practicing safer ways of handling pesticides.

## Discussion

We found that this education-based intervention significantly increased overall knowledge about HCC and its contributing factors among participants from a rural village in Egypt. The increase in participants’ knowledge about the effects of pesticides and aflatoxin in contributing to liver cancer risk was greater than that about HCC and viral hepatitis infections. After the intervention, the participants responded positively when asked about their intention to use best practices to properly store food and reduce exposure to pesticides and to help increase awareness about hepatitis in their community. Notably, health education about these risk factors is the number one recommendation for prevention of HCC, according to the World Gastroenterology Organization’s global guidelines [17].

Our study results are consistent with outcomes of previous community-based approaches to more general cancer prevention in Egypt and elsewhere, such as liver cancer in Pakistan [18], breast cancer in Egypt and Turkey [19, 20], and colorectal cancer in the US [21]. Indeed, Shalaby, et al. reported the results of a study conducted in one of the Egyptian Nile Delta governorates that included a total of 616 participants (308 pairs of barbers and clients) [22]. They showed that 90 % of participants had ever heard of liver disease and 84 % had ever heard of viral hepatitis B and C. Knowledge about the role of blood transfusions and other invasive procedures predisposing to hepatitis B and C virus infection ranged from 68 to 93 %, while knowledge about the role of water and food was only 30 and 39 % respectively [22]. In Pakistan, a significant improvement of knowledge and practices regarding HBV and HCV was observed after an education intervention that involved 70 barbers [18]. A study of HBV and liver cancer among the Asian population in the US concluded that increased health education and improved community-based interventions are necessary to prevent HBV-related liver disease in high-risk groups [23]. In China, where HBV is the primary cause of liver cancer, a total of 1925 students from 26 elementary schools were enrolled in an HBV education program; they achieved significant improvement of knowledge about the modes of transmission and protection against HBV [24].

In our study the most significant gain in knowledge following the educational intervention was in safe use of pesticides and protection measures. Use of pesticides in the household is a common practice in rural areas, yet we found a lack of knowledge about its safe use and measures to reduce exposure. Our finding is consistent with a previous study conducted in a rural Egyptian village in the Nile Delta, in which the knowledge of 335 farmers concerning pesticides was assessed. About 82 % of participants in that study knew that pesticides have a negative effect on health, but only 5 % knew that re-entry to the field following pesticides spraying increases exposure [25].

Some educational interventions have been developed and applied to teach farming families in Egypt how to safely use pesticides [26]. However, these intervention programs do not target liver cancer and its relationship with pesticides use. Our intervention, which specifically stressed the negative relationship between inappropriate use of pesticides and liver cancer risk, resulted in the participants’ affirmative intention to safely use pesticides.

Our study showed some knowledge deficiencies concerning aflatoxin exposure and prevention, with a significant improvement in the knowledge score following the intervention. A similar study conducted in Kenya found that individuals who were exposed to an awareness campaign about aflatoxin had lower serum aflatoxin levels compared to those who were not exposed to the campaign [16]. Another study that was conducted in Benin, Ghana and Togo aimed to increase public awareness about aflatoxin-contaminated maize grains and the adoption of good grain handling practices; the authors concluded that sustained public education could help reduce aflatoxin contamination [27]. Nonetheless, increased knowledge about aflatoxin is necessary but not sufficient in preventing exposure to this HCC risk factor, particularly in countries where food insecurity and climate conditions favoring mycotoxins prevail. Lack of sound knowledge of proper prevention practices may be attributed to the absence or deficiency of health education programs directed towards high-risk groups. Our study showed that intent to use best practices followed the educational intervention. This is consistent with a study conducted in one of the Egyptian Nile Delta governorates, which showed that higher health locus of control scores and higher levels of internal belief among farmers were significantly related to higher scores on health knowledge and behavior scales [25].

The most significant limitations of this study were its relatively small size and the selection of participants from one village in the Nile Delta region. This pilot study was not intended to be generalizable, but rather to serve as the basis for further development of health education interventions in the broader context of community-based participatory prevention research to address HCC. Another limitation is the fact that we were not able to assess actual behavioral changes due to constrained resources. Nonetheless, the results of our study suggest that interest in such prevention research will be high, and that educational interventions are feasible. Future interventions could test this pilot intervention in a large community setting or a large population.

## Conclusion

In conclusion, we found that while there are deficiencies in knowledge concerning HCC and its risk factors among the rural population in Egypt, the education intervention we pilot tested proved effective in raising the knowledge of participants, and most likely resulted in the latter declaring their intent to use best prevention practices. Future efforts should focus on the implementation of targeted education programs in high-risk populations in Egypt and the assessment of practice changes; the ultimate goal being to involve agents of change from the community in educating and empowering individuals, households, and communities as a whole to reduce exposures to biological and chemical carcinogens and achieve cancer prevention and control.
